# Tissue-stiffness–driven platelet activation emerges as a potential target for breast cancer prevention and treatment

**DOI:** 10.1186/s12964-026-03138-x

**Published:** 2026-08-06

**Authors:** Menikae K. Heenkenda, Xingyue Wang, Wen Zhong, Annelie Abrahamsson, Nina Reustle, Daniel Aili, Peter Lundberg, Tomas L. Lindahl, Charlotta Dabrosin

**Affiliations:** 1 Department of Biomedical and Clinical Sciences, Linköping University, Linköping, Sweden; 2 Science for Life Laboratory, Linköping University, Linköping, Sweden; 3 Clinical Department of Oncology, Region Östergötland, Linköping, Sweden; 4 Division of Biophysics and Bioengineering, Department of Physics, Chemistry and Biology, Laboratory of Molecular Materials, Linköping University, Linköping, Sweden; 5 Clinical Department of Medical Radiation Physics, Region Östergötland, Linköping, Sweden; 6 Center for Medical Image Science and Visualization (CMIV), Linköping University, Linköping, Sweden; 7 Clinical Department of Radiology in Linköping, Region Östergötland, Linköping, Sweden; 8 Department of Health, Medicine, and Caring Sciences, Linköping University, Linköping, Sweden; 9 Division of Oncology, Linköping University, Linköping, SE-581 85 Sweden

**Keywords:** Microdialysis, Mammographic density, Platelets, Mechanotransduction, Breast microenvironment, Extracellular signaling

## Abstract

**Background:**

High mammographic breast density is a strong independent risk factor for sporadic breast cancer, yet involved mechanisms remain poorly defined. The extracellular compartment plays a critical role in the intercellular communication during tumor initiation and progression. Although several mechanosensitive pathways have been described, the role of platelets (PLTs) in stiffness-driven signaling in the breast is unknown.

**Methods:**

Extracellular soluble proteins were sampled in situ from live breast tissue using microdialysis. A total of 108 postmenopausal women were included: women with nondense or dense breasts, women with dense breasts randomized to low-dose acetylsalicylic acid (ASA; 160 mg/day) or no treatment, and patients with estrogen receptor–positive (ER^+^) breast cancer. Breast density was assessed by magnetic resonance imaging. High-dimensional proteomic profiling of 1,158 proteins was performed using proximity extension assays. To investigate stiffness-dependent PLT responses, cells were cultured in a 3D in vitro system with tunable matrix stiffness generated by cross-linked hyaluronic acid, modeling nondense and dense breast tissue.

**Results:**

Dense breast tissue exhibited a distinct extracellular proteomic signature enriched for proteins associated with platelet activation, along with alterations in several immunomodulatory pathways. Several PLT-associated proteins were also elevated in ER^+^ breast cancers, supporting their clinical relevance. Post hoc exploratory proteomic analysis of samples from women treated with low-dose ASA did not reveal modulation of these proteins, suggesting that stiffness-induced PLT activation may occur via mechanotransduction rather than biochemical pathways in vivo. Consistently, in the 3D in vitro model, increased matrix stiffness representative of dense breasts promoted a procoagulant PLT phenotype without corresponding changes in classical activation markers.

**Conclusions:**

Tissue stiffness is a critical regulator of PLT mechanotransduction in dense breast tissue, contributing to a microenvironment permissive for cancer progression. These findings highlight PLT mechanobiology as a potential target for breast cancer prevention and therapy.

**Clinical trial registration:**

EudraCT: 2017-000317-22.

**Supplementary Information:**

The online version contains supplementary material available at 10.1186/s12964-026-03138-x.

## Introduction

Breast cancer accounts for approximately 30% of all cancer cases in women in the Western world, and its incidence continues to increase [1]. Although therapeutic advances over recent decades have markedly improved survival, the increasing incidence underscores the urgent need for effective preventive strategies to efficiently decrease disease-related mortality and morbidity. Most breast cancer cases are sporadic, i.e., without any known genetic mutation. One of the most important independent risk factors for sporadic breast cancer is mammographically dense breast tissue [2]. Yet the biological mechanisms linking breast density to increased cancer risk remain poorly understood, and no targeted preventive strategies are currently available.

Women with high mammographic breast density have a 4–6-fold higher risk of developing breast cancer, and women with > 50% dense area are estimated to account for approximately 30% of all breast cancer cases [2]. Several meta-analyses suggest an overall increased risk of breast cancer in relation to breast density regardless of estrogen receptor (ER) status, but the results are more consistent for ER^+^ breast cancer [3–5]. Additionally, there is no difference in the occurrence of ductal carcinoma in situ (DCIS) depending on breast density, which supports the notion that the microenvironment is a key determinant of progression to invasive disease [6]. In normal breast tissue, only 5–10% of the tissue comprises epithelial cells, and there are no conclusive data demonstrating density-related differences in epithelial cell quantity or function [7–9]. The predominant difference lies in the stromal composition in the microenvironment: dense breasts are enriched in collagen, increasing the tissue stiffness, whereas nondense breasts consist largely of adipose tissue, decreasing the tissue stiffness [8]. The importance of the microenvironment for breast cancer development is evident, as a large proportion of women have DCIS, whereas a minority of these cases will eventually progress into invasive breast cancer [10]. Thus, the microenvironment can be more or less permissive for in situ cancer cells to invade the surrounding tissue and progress to invasive breast cancer. In the tissue microenvironment, diverse cell types contribute to cell-cell interactions via the extracellular proteome [11–15]. One important cell type in the microenvironment is platelets (PLTs), which are involved in several pathophysiological processes including cancer progression [16]. Beyond their hemostatic functions, PLTs may affect cancer progression through the release of regulatory proteins and interactions with malignant cells, facilitating both local cancer expansion and metastatic spread via increased angiogenic signaling and immune cell interactions [17, 18]. PLTs may be activated via biochemical or mechanosensitive pathways, and we have previously shown that dense breast tissue is associated with altered coagulation factors and a proinflammatory microenvironment [19–23]. However, a comprehensive understanding of the regulation of the extracellular dynamics and the contribution of PLT signaling in the microenvironment in breast tissue in situ is lacking.

Here, we used microdialysis to sample extracellular proteins directly from live breast tissue of postmenopausal women with mammographically dense or nondense breasts. Breast density was determined as a continuous variable defined by magnetic resonance imaging (MRI). Additionally, microdialysis was also performed in postmenopausal women randomized to low-dose acetylsalicylic acid (ASA) 160 mg/day or no treatment and postmenopausal women with ER^+^ breast cancer. Microdialysates were analyzed using targeted proteomics, quantifying more than 1,100 proteins. A distinct extracellular signature enriched in proteins associated with PLT activation, coagulation, and signaling that strongly correlated with breast density was revealed. A similar protein signature was detected in ER^+^ breast cancers, emphasizing their clinical relevance. Interestingly, a post hoc exploratory analysis of microdialysates after ASA treatment showed no effect on PLT-associated proteins in the breast. In an in vitro 3D model with tunable stiffness representing dense and nondense breast tissue, increased matrix stiffness promoted a procoagulant PLT phenotype. Collectively, our findings provide a comprehensive characterization of the extracellular microenvironment in normal human breast tissues with different densities and suggest that, in addition to immune-related pathways, PLT activation via mechanosensitive pathways also contributes to an extracellular microenvironment with pro-tumorigenic features. These findings identify novel targets that may be used in the development of preventive as well as therapeutic strategies against breast cancer.

## Materials and methods

### Subjects

Previously collected and biobanked samples from different cohorts were used in this exploratory study. The Regional Ethical Review Board of Linköping approved all studies, including biobanking. All studies were carried out in accordance with the Declaration of Helsinki. All subjects gave informed consent.

In the first cohort, healthy postmenopausal women from the mammography screening program at Linköping University Hospital who were categorized according to the Breast Imaging Reporting and Data System (BI-RADS) as either entirely fatty nondense (BI-RADS A) or extremely dense (BI-RADS D) were invited to the study [24]. Forty-three healthy postmenopausal women (ages 55–74 years) were consecutively recruited for the study. The women were also subjected to MRI [22, 25]. As a continuous measure of breast density, lean tissue fraction (LTF) was determined using MRIs as previously described [22, 26]. In brief, a 1.5 T Achieva MR scanner (Philips Healthcare, Best, Netherlands) using a dual breast seven-element breast coil was used. Water- and fat-separated MR images were computed, and the ratio of lean tissue volume to total volume was computed, as previously described [27].

A second review of the mammograms revealed that two women had been miscategorized on the BI-RADS scale. These two women were not included in the analyses of dense vs. nondense breasts but were included in the correlation analyses of LTF.

In the second cohort, biobanked microdialysis samples from women included in a randomized exploratory clinical trial, previously reported [28], were analyzed. In the study, 53 healthy postmenopausal women (55–74 years of age) with dense breast tissue were randomized to acetylsalicylic acid (ASA) 160 mg/day for 6 months or no treatment. Clinical Trial Number EudraCT: 2017-000317-22.

In the third cohort, biobanked microdialysis samples from 12 postmenopausal women with ER^+^ breast cancer that were investigated with microdialysis before surgery were analyzed.

### Microdialysis procedure

Prior to insertion of the microdialysis catheters, 0.5 mL of lidocaine (10 mg*/*mL) was administered intracutaneously. Microdialysis catheters (M Dialysis AB, Stockholm, Sweden), which consisted of a tubular dialysis membrane (diameter 0.52 mm, 100,000 atomic mass cut-off) glued to the end of a double-lumen tube, were inserted via a splitable introducer (M Dialysis AB), connected to a microinfusion pump (M Dialysis AB) and perfused with 154 mmol*/*L NaCl and 60 g/L hydroxyethyl starch (Voluven^®^; Fresenius Kabi, Uppsala, Sweden), at 0.5 µL/min. In the healthy postmenopausal women with various breast densities and in the randomized trial with low-dose ASA or no treatment one 20 mm long microdialysis membrane was placed in the upper lateral quadrant of the left breast directed towards the nipple and another in abdominal s.c fat, as previously described [29–37]. In the women with breast cancer, 10 mm long membranes were inserted within the breast cancer and another within adjacent normal breast. After a 60-min equilibration period, the outgoing perfusate was stored at -80 °C for subsequent analysis. In Supplementary Figures, a schematic illustration of the procedure is depicted.

### Protein quantifications

Microdialysates were analyzed using multiplex proximity extension assay (PEA, Olink Bioscience, Uppsala, Sweden) as previously described [21, 23, 38]. A total of 1,158 unique proteins in 13 panels, including Cell Regulation, Cardiovascular II, Cardiovascular III, Inflammation, Cardiometabolism, Development, Organ Damage, Neurology, Neuro Exploratory, Immune Response, Metabolism, Oncology II, and Oncology III (Olink Bioscience, Uppsala, Sweden), were quantified. In brief, 1 µL of sample was incubated with proximity antibody pairs tagged with DNA reporter molecules. The DNA tails formed an amplicon by proximity extension, which was quantified by high-throughput real-time PCR (BioMark™ HD System; Fluidigm Corporation, South San Francisco, CA, USA). The generated fluorescent signal correlates with protein abundance by quantitation cycles (Cq) produced by the BioMark Real-Time PCR Software. Data were normalized using both an internal control (extension control) and an interplate control and transformed using a predetermined correction factor. The pre-processed data were provided in the arbitrary unit normalized protein expression (NPX) on a log_2_ scale, which were then linearized by using the formula 2^NPX^. A high NPX value corresponds to high protein concentrations. Values represent a relative quantification, meaning that no comparison of absolute concentrations between different proteins can be made.

### Estradiol analysis

Estradiol levels were analyzed using a high-sensitivity immunoassay kit (DRG International, Springfield Township, NJ, USA).

### Cells

The ER⁺ breast cancer cell line MCF-7 (ATCC, HTB-22; RRID: CVCL_0031) was verified by short tandem repeat profiling at the Uppsala Genome Center. Cells were cultured in Dulbecco’s Modified Eagle Medium (DMEM; Gibco, Cat# 11880) supplemented with 2 mM L-glutamine (Gibco, Cat# 25030), 50 IU/mL penicillin-G, 50 µg/mL streptomycin (Gibco, Cat# 15070), and 10% fetal bovine serum (FBS; Gibco, Cat# 10270).

PLTs were isolated from venous blood obtained from healthy female donors. Blood was collected into acid–citrate–dextrose (ACD) tubes (Vacuette, Greiner Bio-One, Austria). Immediately upon collection, apyrase (0.5 U/mL; Sigma-Aldrich, MO, USA, Cat#A6535) and prostaglandin-E1 (PGE_1_, 100 nM; Sigma-Aldrich, MO, USA, Cat#P5515) were added, and the samples were incubated for 30 min at RT. PLT-rich plasma (PRP) was generated by centrifugation at 150 × g for 15 min. The PRP was carefully aspirated and supplemented with apyrase (0.5 U/mL). PLTs were pelleted by centrifugation and washed in Krebs–Ringer–Glucose (KRG) buffer (120 mM NaCl, 4.9 mM KCl, 1.2 mM MgSO₄, 1.7 mM KH₂PO₄, 8.3 mM Na₂HPO₄, and 10 mM glucose; pH 7.3; Sigma-Aldrich, MO, USA), which was slowly added to minimize shear-induced PLT activation. For the second wash, KRG was supplemented with apyrase (0.5 U/mL) and PGE_1_ (100 nM). The PLT pellet was resuspended in inhibitor-containing KRG to a final concentration of 8 × 10⁸ PLT/mL.

### Hydrogels

Hyaluronic acid (HA) was functionalized with bicyclo [6.1.0]non-4-yne (BCN), and HA-BCN hydrogels were formed in serum-free medium by strain-promoted azide–alkyne cycloaddition (SPAAC) with 4-arm PEG-azide (PEG-Az4), maintaining a constant BCN-to-azide ratio of 2.6, as previously described [39–41]. Hydrogel mechanical properties, stability, and mesh size were assessed by oscillatory rheology, swelling analysis, and nanoparticle diffusion assays [41].Depending on whether shear-based or elastic methods are used to measure breast stiffness, a wide range of tissue stiffness values has been reported for nondense and dense breasts. Reported values range from 0.2 to 1 kPa for nondense breast tissue and from 0.7 to 2.4 kPa for dense breast tissue [42–45]. However, when stiffness is quantified using Young’s modulus (defined as force per unit area relative to the resulting deformation), the most frequently reported in vivo values are approximately 0.2 kPa for nondense breast tissue and 0.8–0.9 kPa for dense breast tissue [45]. Accordingly, we prepared hydrogels within these stiffness ranges to mimic the two tissue stiffness conditions.

### 3D co-culture of PLTs and breast cancer cells

MCF-7 cells were suspended in serum-free DMEM/F12 medium (Gibco, Cat#11039) supplemented with 0.02% bovine serum albumin (Merck, Cat#1.12018.0025), 10 µg/mL apo-transferrin (Sigma, Cat#T2036), 1 µg/mL insulin (Sigma, Cat#I5500), and 1% penicillin–streptomycin (Gibco, Cat#L0022-100). MCF-7 cells and freshly isolated PLTs, at a ratio of 1:40, were suspended in HA-BCN at 14.87 mg/mL or 10.5 mg/mL and 50 µg/mL fibrinogen (Sigma-Aldrich, St. Louis, MO, USA, Cat# F3879). The cross-linker PEG-Az4 was added to generate hydrogels with different stiffnesses, median (25th–75th percentile) of 0.2 kPa (0.18–0.24) representing nondense breasts and 0.85 kPa (0.72–0.95) representing dense breasts, respectively. Ten microliters were dispensed in µ-Slide 15-well chambers (Ibidi GmbH, (RRID: SCR_027508) and allowed to polymerize for 1 h at 37 °C in a humidified incubator (5% CO₂, 99% humidity). After polymerization, 50 µL of serum-free medium, ± PAR1-activating peptide (PAR1-AP; JPT Peptide Technologies GmbH, Berlin, Germany), ± ROCK inhibitor Y-27632 (10 µM; Sigma-Aldrich, St. Louis, MO, USA) was added, and co-cultures were incubated for 2–24 h.

### Immunofluorescence staining of 3D cultures

Samples were washed twice with Ca²⁺-containing PBS. Activated integrin αIIbβ3 was detected using PAC-1-AF405 mouse monoclonal IgM κ antibody (Novus Cat# NBP2-62201AF405, RRID: AB_3352199), and phosphatidylserine exposure was assessed using recombinant human Annexin V-AF647 (Invitrogen, Cat# A23204; RRID: AB_2341149; 1:20). Reagents were diluted in Ca²⁺-supplemented HEPES buffer. Samples were incubated for 45 min at 37 °C and then washed three times with Ca²⁺-containing PBS, fixed with 2% buffered formalin for 15 min at RT, and blocked with 2% BSA in PBS for 1 h with shaking. The total number of PLTs was identified using CD41-AF488 mouse monoclonal IgG1 antibody (Thermo Fisher Scientific Cat# MA5-18137, RRID: AB_2539511), 1:20 and α-granule secretion was assessed using P-selectin-AF700 mouse monoclonal IgG1 antibody (Novus Cat# NB100-65392AF700, RRID: AB_3168537, 1:150). Antibodies were diluted in background-reducing antibody diluent (Agilent/DAKO, Glostrup, Denmark; Cat# S302281-2) and incubated for 2 h with shaking. Samples were permeabilized with 1% Triton X-100 for 10 min. F-actin was stained with rhodamine-phalloidin (Thermo Fisher Scientific Cat# R415, RRID: AB_2572408, 1:400) for 20 min. Samples were washed in PBS containing 1% Tween-20 and imaged by confocal microscopy.

### Confocal microscopy acquisition

Fluorescence imaging was performed using an inverted Nikon Eclipse Ji spinning disk confocal microscope (Nikon Instruments, Tokyo, Japan). Z-stack images were acquired using Nikon imaging software (NIS-Elements RRID: SCR_014329) with laser excitation at 405, 477, 545, 637, and 748 nm, selected according to the fluorophores used. Images were captured at 12-bit depth with no pixel binning and a frame size of 2048 × 2048 pixels. Exposure times and laser power were kept constant within each experiment. Multichannel images were acquired sequentially using appropriate emission filters. All imaging parameters were identical across experimental conditions to allow quantitative comparison.

### Image quantification and analysis

Image analysis was performed using ImageJ/Fiji (RRID: SCR_002285). Z-stacks were converted to maximum-intensity projections and separated into individual channels. PLTs were segmented based on CD41 immunofluorescence following median filtering (radius = 2 pixels) and manual global thresholding. Individual PLTs were identified by particle analysis (size 1–35 μm²; circularity 0–1.0), excluding edge-touching objects. The resulting PLT regions of interest (ROIs) were applied uniformly across all marker channels. Annexin V, PAC-1, and P-selectin channels were denoised, background-subtracted, thresholded, and intersected with the CD41 mask using logical AND operations. Marker-positive PLTs were quantified using identical size and circularity criteria and normalized to total CD41-positive PLTs per field.

### Statistical analyses

Statistical analyses were performed using two-sided unpaired Mann-Whitney U tests, Student’s t-test, Wilcoxon signed-rank test for paired observations, one way ANOVA with Bonferroni’s post hoc test, and Spearman’s correlation test where appropriate. A *P* < 0.05 was considered statistically significant. False discovery rate (FDR) was performed using the two-stage set-up method of Benjamini, Krieger, and Yekutieli, which was set at 5%.

Gene ontology (GO) enrichment analysis (biological process) and Reactome pathway enrichment analysis were performed based on the significant assays (P-value < 0.05). Significant protein-protein associations were identified based on Spearman’s correlation coefficients and p-values, using thresholds of |R| > 0.8 and FDR < 1e^− 10^ for the comparison of dense versus nondense groups. In the association analyses, one sample or measure for each participant was used. Protein-protein interaction network analysis incorporated 568 protein pairs involving 132 proteins. The constructed protein-protein networks were weighted and undirected, with edge weights reflecting the absolute values of the correlation coefficients. The Louvain algorithm was applied for community analysis. Key centrality metrics, including degree, betweenness, and closeness, were calculated for each protein. Network analysis and visualization were performed using the R package igraph v2.0.3 and qgraph v1.9.8. GO and KEGG enrichment analysis and visualization for protein communities were conducted using R packages clusterProfiler v4.10.1 and ggplot2 v3.5.1, with the 1,158 proteins quantified in the study used as the background. (R version 4.3.3). All statistics were performed with Prism 10.0 (GraphPad, San Diego, CA, USA) and R version 4.2.2 (2022-10-31).

## Results

### Characterization of the subjects

Characteristics of the three patient cohorts are included in Supplementary Tables S1-3. In the cohort of women with dense or nondense breasts, no differences in body mass index (BMI), age, or local breast estradiol were detected. There was a significant difference in LTF between dense and nondense breast tissue. In the ASA or no treatment cohort, no differences in body mass index (BMI), age, or initial LTF were detected. In the cohort of women with breast cancer, age, tumor size, grade, and receptor characteristics are included in Supplementary Table S3.

### Expression profile of proteins in dense versus nondense breast tissue

**Fig. 1:**
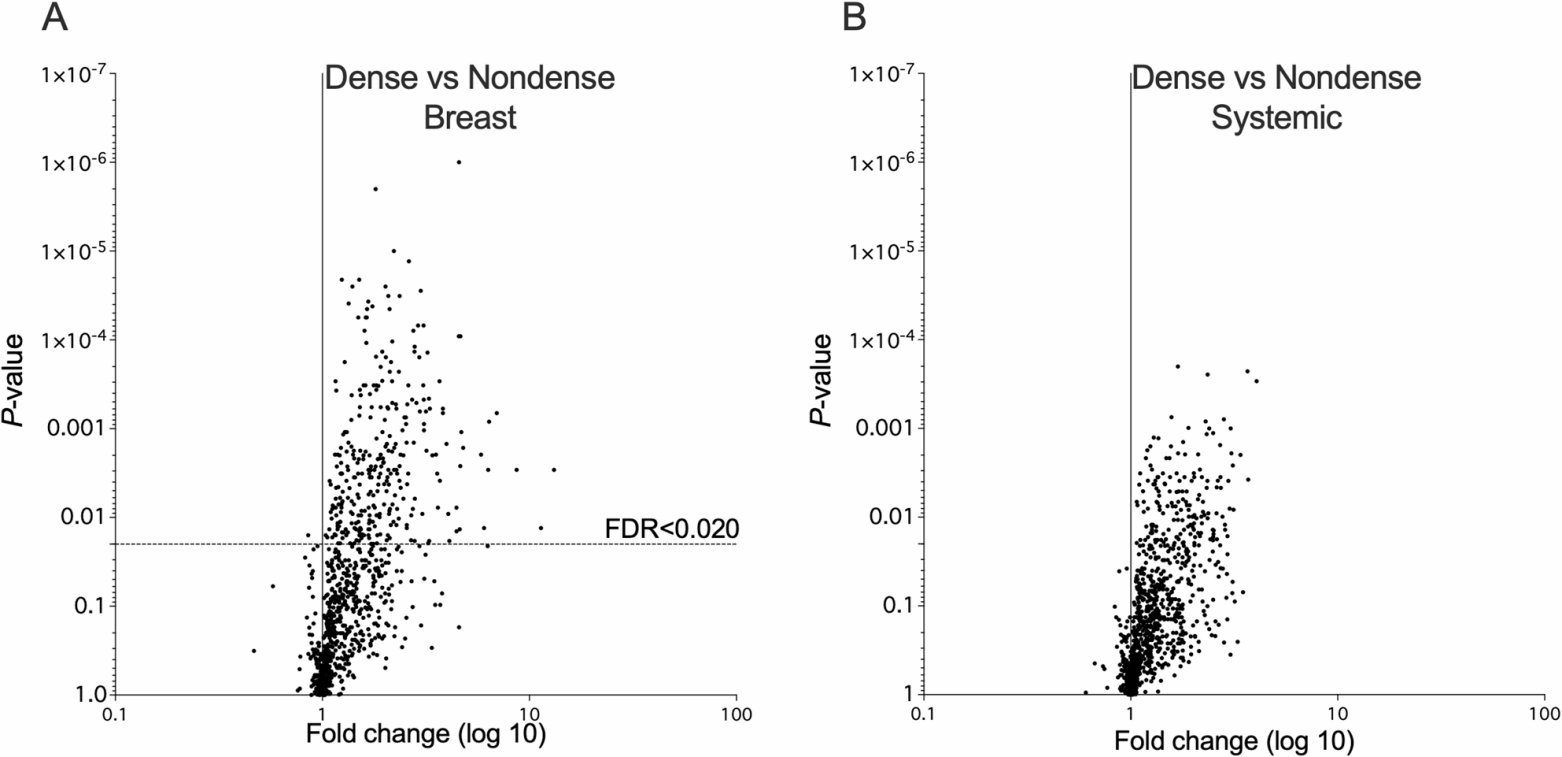
In situ extracellular protein signatures systemically and in normal breast tissue with different densities in postmenopausal women. Microdialysis for in vivo sampling of local extracellular proteins was performed in breast tissue and abdominal subcutaneous fat, as a proxy for systemic alterations, of postmenopausal women with different mammographic breast densities (*n* = 20 in the nondense (BI-RADS A) and *n* = 21 in the dense group (BI-RADS D)). Proximity extension assay targeting 1,158 individual proteins in the microdialysates quantified 984 proteins above the lowest level of detection (LOD) in > 50% of the samples in at least one group . **A** Profile of extracellular proteins in normal breast tissues. **B** Proteins in abdominal subcutaneous fat from the same participants as in (**A**). FDR = False Discovery Rate

A total of 984 out of 1,158 proteins were above the lower level of detection (LOD) in > 50% of the samples in at least one group. After FDR correction, a total of 307 proteins were significantly changed in dense breast tissue vs. nondense breast (Fig. 1A). As a proxy for systemic changes, we sampled proteins with the same technique, i.e., microdialysis from abdominal s.c. fat. This approach allows for distinguishing local breast tissue changes from systemic differences. As shown in Fig. 1B, the profile of the proteins in abdominal s.c. fat from women with dense vs. nondense breast tissue was skewed similarly to the one observed in breast tissue. However, no significant changes were detected after FDR correction.

### Breast tissue-specific and systemic differences

We next identified proteins with breast tissue–specific alterations by comparing significantly altered proteins in the breast with those in abdominal s.c. adipose fat. Microdialysis of abdominal s.c. fat was used as a proxy for systemic alterations rather than blood, as it is important to sample proteins from the same compartment and within the same matrix, i.e., tissue extracellular fluid, when comparing protein levels. In principle, the microdialysis catheter could have been placed in any other anatomical site. However, during the development of the microdialysis technique for human use, abdominal s.c. fat was established as a standard site for systemic monitoring [46]. Based on this precedent, as well as for practical considerations, we used abdominal s.c. fat as a proxy for systemic protein alterations. By comparing these two sets of proteins, breast tissue and abdominal s.c. fat, 206 proteins were found to be significantly changed in breast tissue only, 101 proteins were changed in both breast tissue and fat, and 60 proteins changed in fat tissue only (Supplementary Table S4).

### Co-expression network of breast tissue-specific proteins

**Fig. 2:**
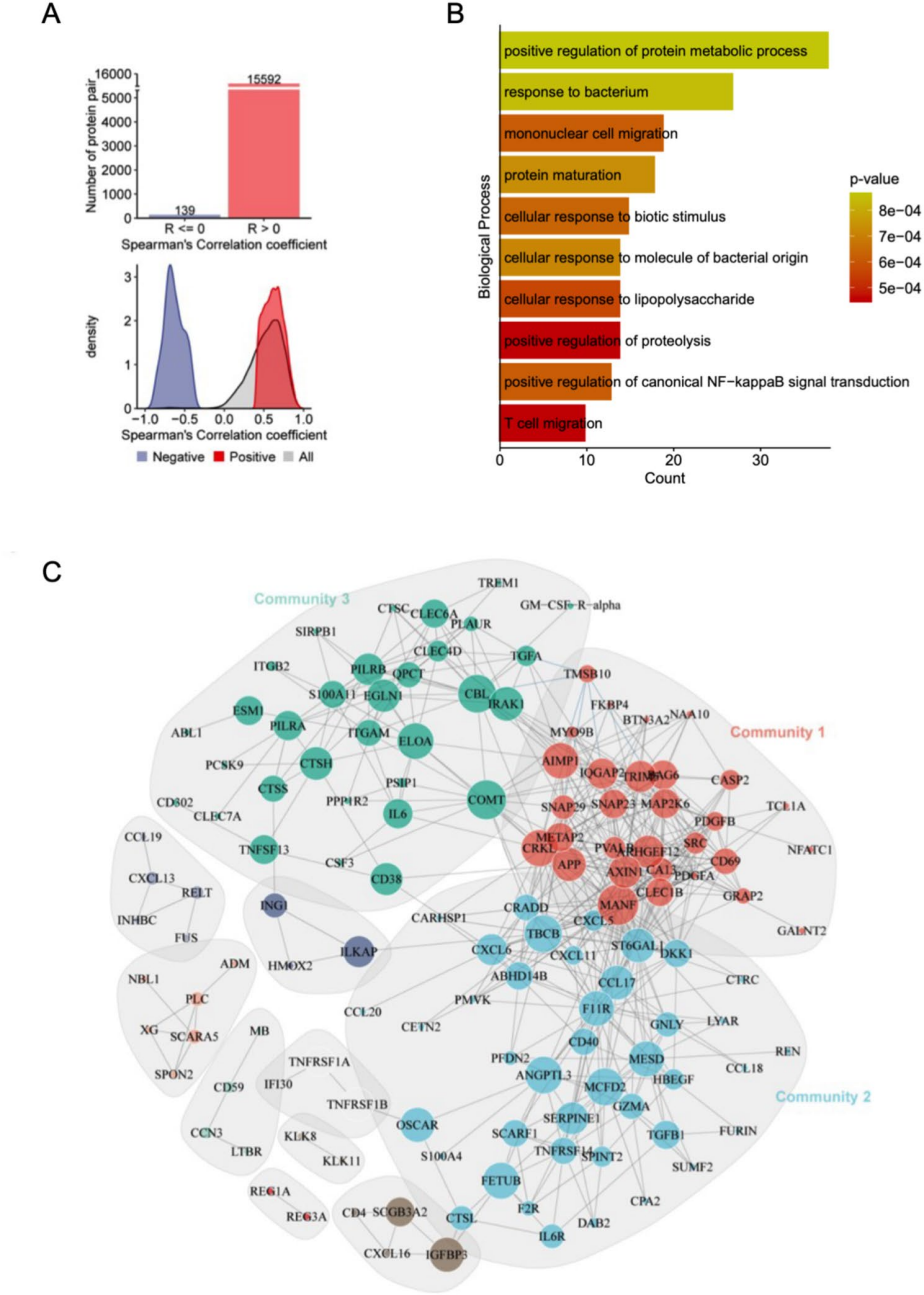
Co-expression pattern of breast tissue-specific proteins in postmenopausal women with dense or nondense breasts. Women were investigated with microdialysis as described in Fig. 1. **A** Bar plot showing the number of positive and negative associations (FDR < 0.01) between the 206 breast tissue-specific proteins comparing postmenopausal dense versus nondense breast tissue. Density plot showing the distribution of Spearman correlation coefficients, with the distribution of negative correlations, positive correlations, and all the associations colored blue, red, and grey, separately. **B** Bar plot showing Gene Ontology-Biology Process enrichment analysis result of the 132 proteins with the most significant correlations (FDR < 1e^− 10^, |R|<0.8). **C** A protein-protein association network based on the 568 associations between 132 proteins, with the different communities circled by grey lines and nodes colored by the community they belonged to. The node size is positively correlated with the betweenness score. FDR = False Discovery Rate

To further investigate the co-expression pattern of breast tissue-specific proteins, we performed Spearman correlation analysis on the 206 breast-specific altered proteins in dense vs. nondense groups. Out of 21,115 protein pairs between 206 proteins analyzed, 15,731 protein pairs had an FDR < 0.01, with more than 99% of the correlations being positive. Only 139 negative correlations were observed, primarily between TMSB10 (thymosin beta 10, a serum marker of breast cancer) and other proteins (Fig. 2A). Among these protein pairs, 568 pairs between 132 proteins had FDR < 1 × 10^− 10^ and an absolute value of correlation coefficient (*|R|*) larger than 0.8. Enrichment analysis showed that most of them were involved in the regulation of metabolic processes and signaling receptor binding (Fig. 2B). A protein-protein association network was built based on these 132 proteins, with 12 communities detected. Three closely related communities were mainly involved in the Rap1 signaling pathway (community 1) and cytokine-cytokine receptor interaction (community 2). The most significant association was found between PDGFB (PLT derived growth factor subunit B) and SRC (proto-oncogene tyrosine-protein kinase Src) (*R* = 0.97, FDR = 1.53e^− 27^) (Fig. 2C).

Overall pathway analyses in the breast without considerations of breast-specific alterations are shown in Supplementary Fig. S1A-D.

Importantly, protein-protein correlation analyses identify co-expression networks regardless of protein associations with breast density determined using LTF.

### Correlations with breast density and levels of individual proteins with the highest correlation coefficient

**Fig. 3:**
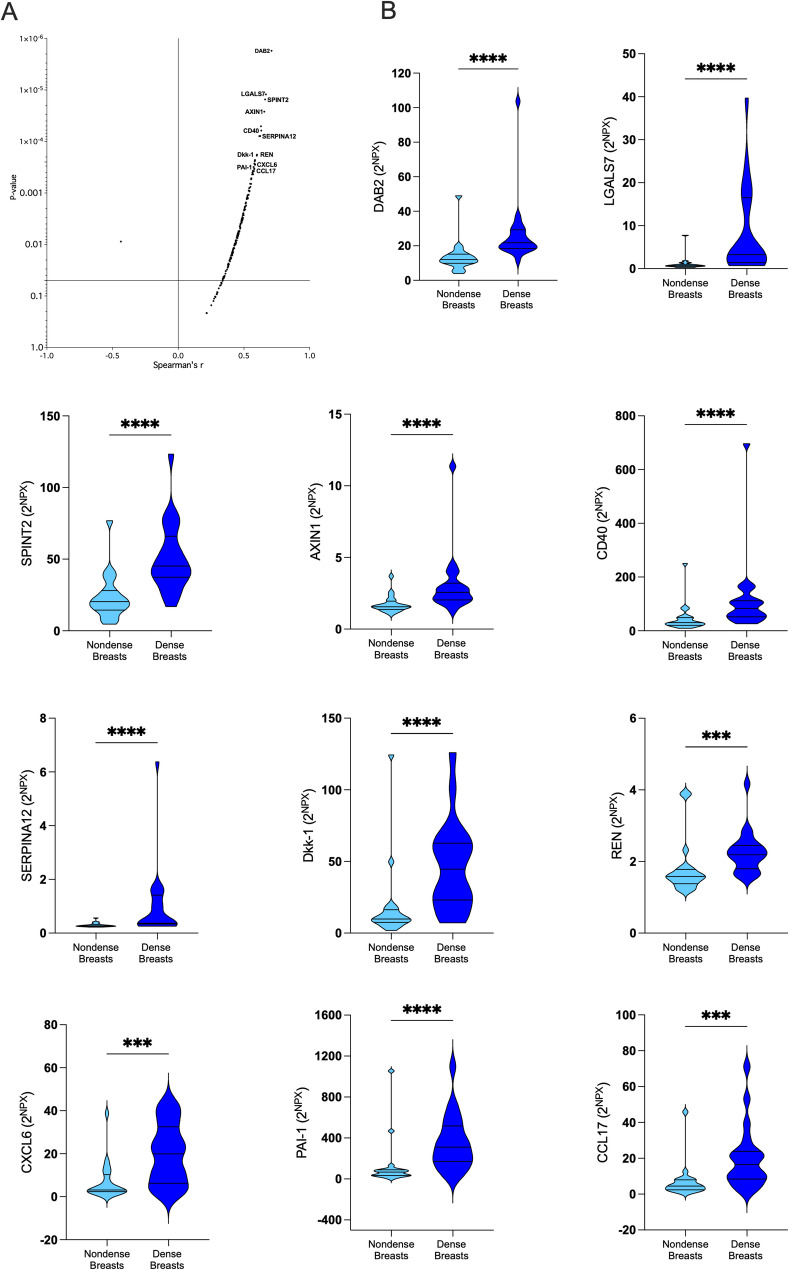
Correlations of breast tissue-specific altered proteins and breast density measured by lean tissue fraction (LTF). Extracellular proteins were sampled by microdialysis as described in Fig. 1. **A** Spearman correlation coefficients for the associations between proteins that were altered in dense breast tissue only and breast density expressed as LTF determined by magnetic resonance imaging. **B** Levels of the proteins with the strongest correlations with breast LTF that are associated with PLT activation and function. Violin plots with median and quartiles, Mann–Whitney test, ***P < 0.001, ****P < 0.0001

To further explore the relationships of protein levels to breast density, we analyzed whether the breast-specific proteins correlated with LTF. We found that 186 of the 206 breast-specific proteins exhibited a significant positive correlation with LTF, whereas one protein exhibited a negative correlation (Fig. 3). Out of the 20 proteins with the highest correlation coefficients with LTF, eleven have been shown to be directly or indirectly involved in PLT activation, highlighted in Fig. 3A. In Fig. 3B, the levels of the 11 proteins associated mainly with PLT activation and coagulation are depicted. SRC and PDGFB were not among the proteins with the most significant correlation coefficient with LTF; in fact, 78 and 87 proteins, respectively, showed higher correlations with LTF. As the primary aim of this study was to elucidate the role of LTF in microenvironmental signaling, subsequent analyses were therefore focused on proteins exhibiting the highest correlations with LTF.

### Proteins upregulated in human ER^+^ breast cancer

**Fig. 4:**
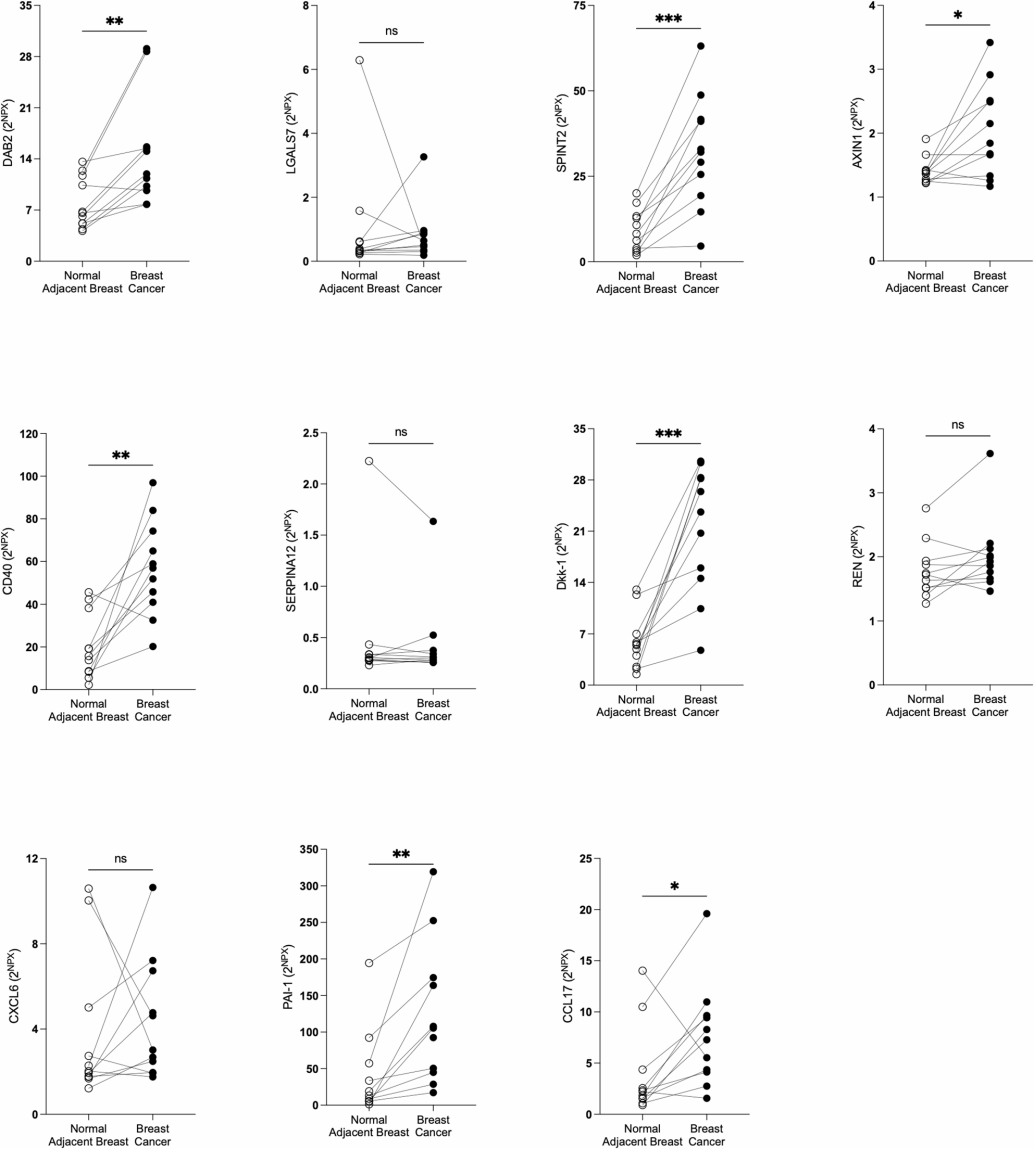
Increased levels of platelet-associated proteins in human ER^+^ breast cancer. Extracellular proteins in breast cancers and adjacent normal breast tissues were sampled in situ by microdialysis in postmenopausal patients with ER^+^ breast cancer before surgery. Microdialysates were quantified as described in the Materials and Methods. Wilcoxon’s signed rank test for paired observations, ns = not significant, *P < 0.05, **P < 0.01, ***P < 0.001

Next, we investigated whether the upregulated proteins associated with LTF were increased in human ER^+^ breast cancer. As shown in Fig. 4, 7 of the proteins, DAB2, SPINT2, AXIN1, CD40, Dkk-1, PAI, and CCL17, exhibited increased levels within the cancer as compared to normal adjacent breast tissue.

### No effects of ASA on the proteins with the highest correlation with LTF

**Fig. 5:**
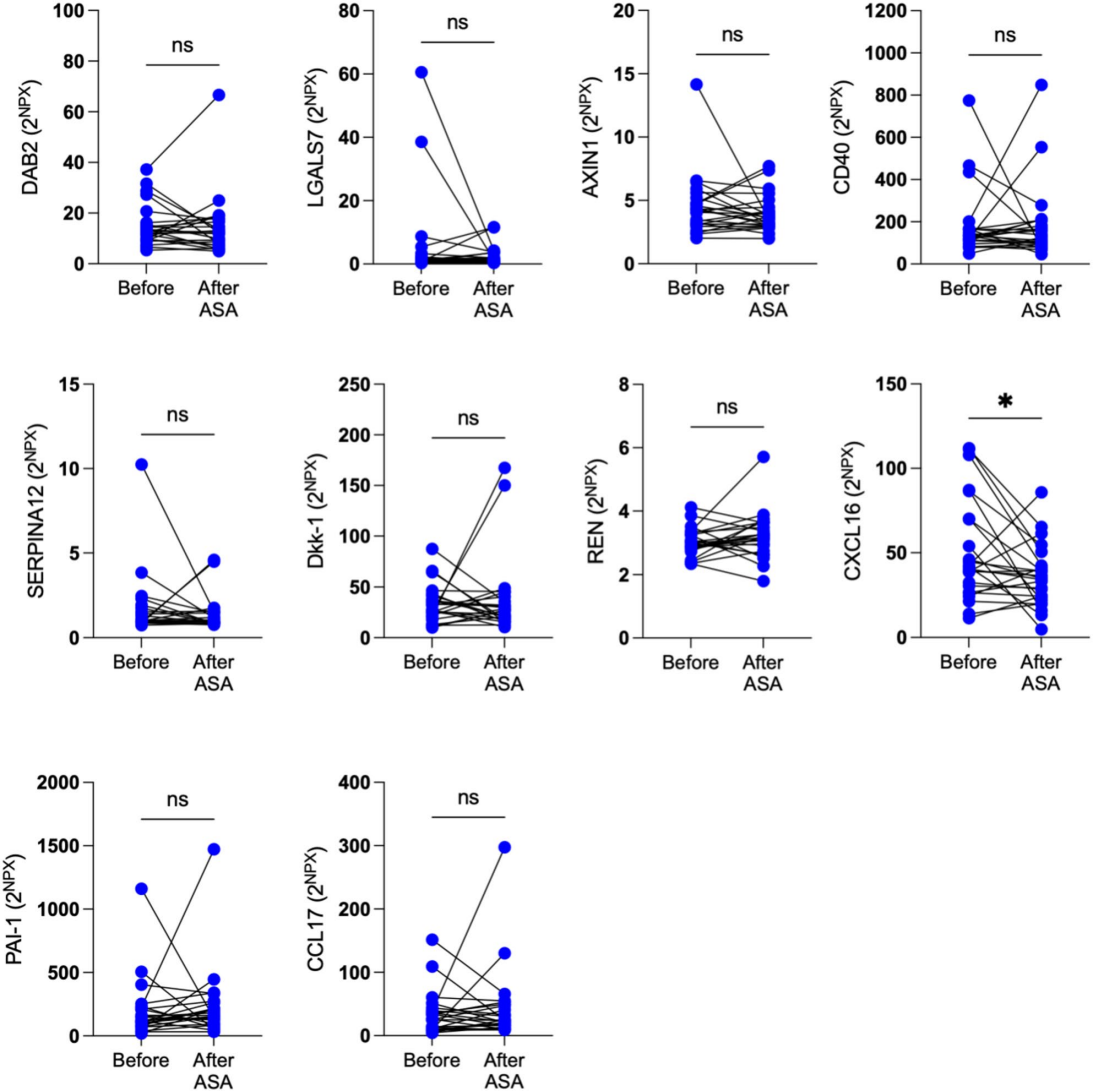
Effects of low-dose ASA on proteins upregulated in dense breasts. Postmenopausal women with dense breasts were treated with low-dose ASA 160 mg daily for six months. Microdialysis for sampling of extracellular proteins in the breast was performed before and after therapy, (n = 24). Wilcoxon’s signed rank test for paired observations, ns = not significant, *P < 0.05

Thereafter, we performed a post hoc exploratory analysis of data from a randomized clinical trial where postmenopausal women with dense breast tissue were treated with low-dose ASA or no treatment for six months. Two women in the ASA group interrupted treatment due to skin rash, and in the no-treatment group, two women started regular non-steroidal anti-inflammatory drugs (NSAIDs) for medical reasons; thus, 25 women in the ASA group and 24 in the no-treatment group were subjected to microdialysis both before and after six months. We have previously reported a significant decrease in the levels of several proinflammatory proteins after ASA therapy for six months, whereas no difference in breast density was detected [28]. ASA exerts effects on coagulation and PLT function via biochemical pathways. To elucidate whether the proteins that were increased in dense breasts were affected by ASA, we analyzed biobanked microdialysates from the clinical trial. As shown in Fig. 5, only CXCL16 was affected by low-dose ASA, whereas the other PLT-related proteins that correlated with LTF were unaffected. There were no changes of any protein level in the no-treatment group, data not shown.

### Increased matrix stiffness promoted a procoagulant PLT phenotype

**Fig. 6:**
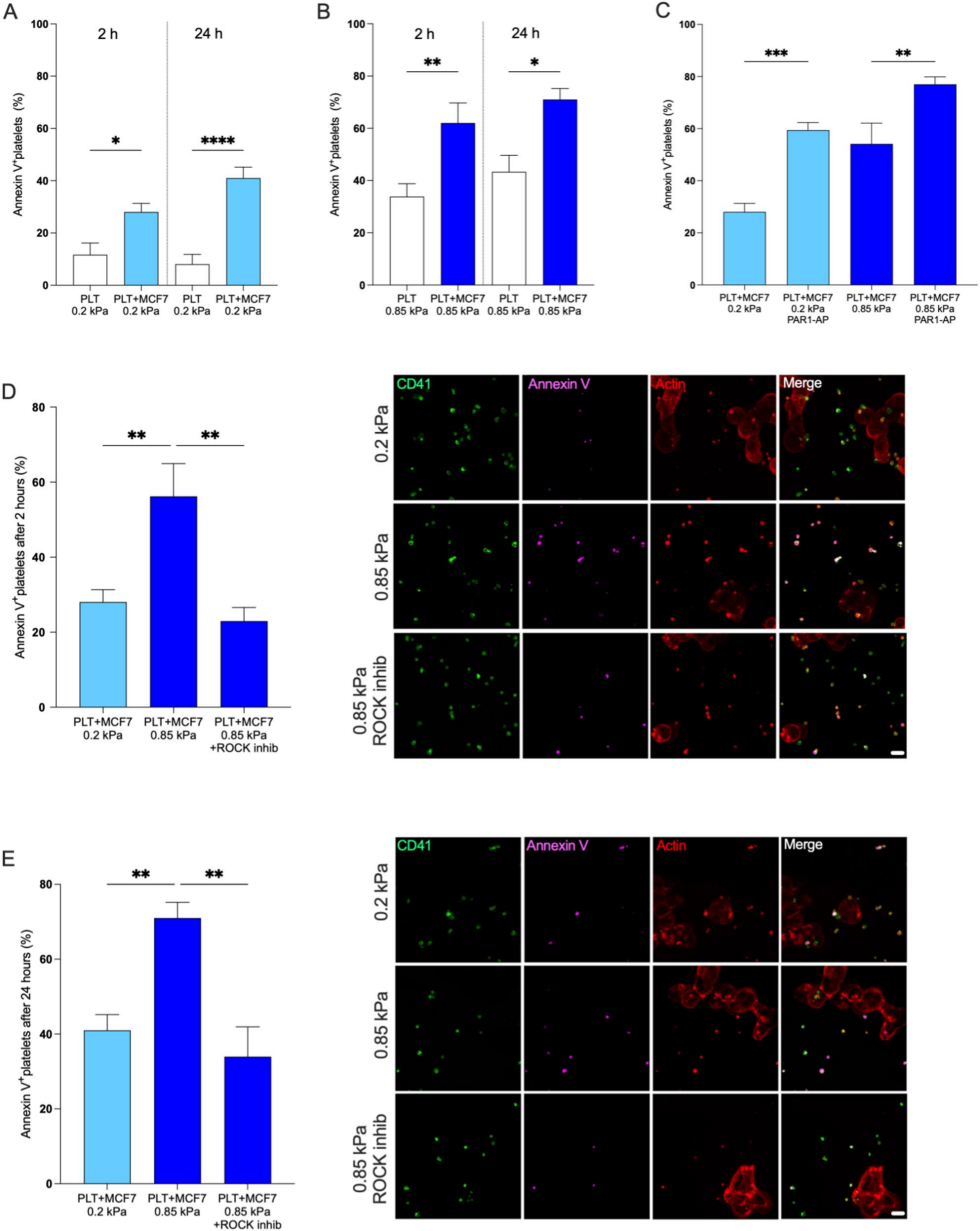
High matrix stiffness is associated with increased platelet (PLT) phosphatidylserine exposure in co-culture with breast cancer cells. Human PLTs were cultured alone or together with estrogen receptor-positive MCF-7 breast cancer cells in 3D HA-BCN hydrogels of low (0.2 kPa) or high (0.85 kPa) matrix stiffness and analyzed after 2–24 h. PLT phosphatidylserine exposure was quantified by Annexin V binding. ROCK was inhibited with Y-27,632 as described in the materials and methods section. **A** Annexin V-positive PLT cultured ± MCF-7 cells in 0.2 Pka after 2 and 24 h. Student’s t-test, *n* = 4–6 in each group, *P < 0.05, ****P < 0.0001. **B** Annexin V-positive PLT cultured ± MCF-7 cells in 0.85 kPa after 2 and 24 h. Student’s t-test, *n* = 4–6 in each group, *P < 0.05, **P < 0.01. **C** Annexin V-positive PLT after 2 h of co-culture with MCF-7 in ± PAR1 activation (PAR1-AP), Student’s t-test, *n* = 6–7 in each group, **P < 0.01, ***P < 0.001. **D** Annexin V-positive PLTs after 2 h of co-culture with MCF-7 ± ROCK inhibitor. One-way ANOVA with Bonferroni’s post hoc test, *n* = 6–7 in each group, **P < 0.01. Representative confocal images. **E** Annexin V-positive PLTs after 24 h of co-culture with MCF-7 ± ROCK inhibitor. One-way ANOVA with Bonferroni’s post hoc test, *n* = 4 in each group, **P < 0.01. Representative confocal images show PLT identified by CD41 staining (green) and phosphatidylserine exposure detected by Annexin V binding (magenta). MCF-7 cancer cells are visualized by F-actin staining using rhodamine phalloidin (red). Scale bar = 10 μm

ASA inhibits PLT activation mainly via biochemical pathways by blocking cyclooxygenase and thromboxane A2 synthesis. However, as PLTs also can respond to mechanical cues, we next investigated whether matrix stiffness could alter PLT behavior under controlled in vitro conditions, independently of canonical cyclooxygenase-mediated pathways. We set up 3D gels with tunable stiffness, where 0.85 kPa resembles dense breasts and 0.2 kPa nondense breasts. In the first set of experiments, we identified an appropriate matrix ligand. cRGD-containing hydrogels exhibited reduced PLT association with the matrix, and collagen type I induced immediate PLT activation, masking stiffness-dependent effects, whereas fibrinogen-containing hydrogels exhibited stable PLT association and allowed stiffness-dependent activation (Supplementary Fig. S2). Therefore, we used fibrinogen as a matrix ligand for the subsequent experiments. In the first experiment, we explored the performance of PLTs cultured alone or together with MCF-7 breast cancer cells. Co-culture of PLTs with MCF-7 cells significantly increased Annexin V expression as compared to PLTs cultured alone after 2 h and 24 h in both stiffness conditions (Fig. 6A-B). After 24 h, PLT cultured alone in 0.2 kPa hydrogels showed a reduced Annexin V response compared to 2 h. In contrast, co-culture with MCF-7 cells resulted in a sustained increase in the proportion of Annexin V-positive PLTs at both time points and across both hydrogel stiffnesses. Therefore, to investigate the behavior of PLT within a tumor-like microenvironment and in interaction with atypical breast cells, all subsequent experiments were performed under co-culture conditions. Next, we examined whether PLTs remained functionally responsive under these conditions. PAR1 activation significantly increased the proportion of Annexin V-positive PLTs in both low- and high-stiffness hydrogels (Fig. 6C), indicating preserved functional responsiveness of PLT in co-culture. To investigate whether stiffness-dependent PLT activation involved cytoskeletal regulation, ROCK, a key regulator of actomyosin contractility, was inhibited. The increased Annexin V exposure observed after 2 h in 0.850 kPa hydrogels was significantly reduced by ROCK inhibition (Fig. 6D), indicating mechanosensitive regulation of PLT phosphatidylserine exposure. This stiffness-dependent effect was maintained over time, as similar results were observed after 24 h (Fig. 6E). Representative confocal images at 2 h and 24 h are shown in Fig. 6D-E. Notably, matrix stiffness did not induce a corresponding increase in classical PLT activation markers, as no significant changes in surface expression of P-selectin or PAC-1 were detected under either stiffness condition or time point (Supplementary Fig. S3), suggesting a procoagulant PLT phenotype rather than classical platelet activation.

## Discussion

Here, we showed that proteomic profiling of dense breast tissue revealed a distinct molecular signature in situ enriched for proteins associated with PLT function, coagulation, and related signaling pathways. The proteins that were upregulated in dense breasts were also increased in ER^+^ breast cancer, emphasizing the clinical relevance of these proteins. Interestingly, low-dose ASA did not affect local breast tissue levels of the proteins related to PLT activation. Our in situ data were corroborated in an in vitro 3D model with tunable stiffness resembling that of dense and nondense breast tissue. Increasing matrix stiffness representing dense breast tissue promoted a procoagulant PLT phenotype. Furthermore, consistent with previous reports, we also demonstrate here that several immune-related signatures, including CCLs, CXCLs, and interleukins, were elevated in dense breast tissue [21, 22, 47]. The combination of this pro-inflammatory microenvironment with PLT activation may further shift the microenvironmental balance toward a pro-tumorigenic state in dense breasts.

During homeostasis, PLTs circulate in the bloodstream and will only accumulate in injured tissue where they become activated upon exposure to extracellular matrix (ECM) components, such as collagen. We and others have previously shown that dense breast tissue is associated with a local proinflammatory microenvironment, including increased levels of inflammatory and angiogenic factors [21, 22, 48–50]. This milieu promotes increased vascular permeability through disruption of the endothelial barrier, and MRI studies have demonstrated that dense breasts indeed are characterized by altered perfusion and increased edema, supporting physiological effects by the biological findings [47]. A further physiological consequence is enhanced extravasation of immune cells, as previously reported in dense breasts [22, 50]. Thus, the disruption of the endothelial barrier in dense breast tissue may also facilitate PLT leakage into the microenvironment, which was confirmed in this study by immunostaining of normal human breast tissue (Supplementary Fig. S4).

The cell-cell interactions in the microenvironment are largely a result of signaling mediated by soluble proteins. Microdialysis, which we have used in this study, enables sampling of soluble proteins directly from live tissue. With our experimental setup of using microdialysis of abdominal s.c. fat as a control, we could delineate breast tissue-specific alterations, as all proteins were collected with the same method and in the same matrix as in the breast.

Of the 984 proteins that were quantified, we could identify 206 proteins that were significantly altered in breast tissue only. Enrichment was observed in pathways such as cytokine-cytokine receptor interactions, as well as in signaling pathways including the chemokine signaling pathway and the ErbB signaling pathway.

To further distinguish the role of breast density on protein regulation, correlation analyses with LTF, a continuous measure of density determined using MRI, were performed. Of the 206 breast tissue-specific proteins, 186 correlated significantly with LTF. Several proteins with the highest correlation coefficients, including DAB2, LGALS7, SPINT2, AXIN1, CD40, SERPINA12, Dkk-1, CXCL6, PAI-1, and CCL17, are associated with platelet function, coagulation, and related signaling pathways.

DAB2 is a key regulator of PLT signaling and activation [51]. LGALS7 belongs to the galectin family of proteins that induce aggregation of PLT [52]. SPINT2 affects blood coagulation and fibrinolysis by inhibiting human plasmin, kallikrein, and factor Xia, resulting in prolonged clotting time [53]. AXIN1 is overexpressed and associated with Wnt signaling in PLT and epithelial cells [54, 55]. CD40 is expressed on PLT, and when bound to its ligand, coagulation and activation of inflammatory pathways are induced [56]. SERPINA12 is associated with treatment-related PLT reactivity during anti-thrombotic agents [57]. Classically, REN is produced by the kidney. However, local tissue REN has also been discovered and shown to be involved in various pathways maintaining local homeostasis, including tissue inflammation, fibrosis, hemostasis, and coagulation, by inducing a prothrombotic state via the renin-angiotensin system [58, 59]. Activated PLT release Dkk1, which in turn may activate endothelial cells and the release of inflammatory cytokines [60]. CXCL6 triggers PLT activation, degranulation, and shape change [61]. PLTs synthesize PAI-1, which is released upon PLT activation. PAI-1 acts as a procoagulant by inhibiting tissue plasminogen activation (tPA) through the formation of an inactive complex, thereby suppressing fibrinolysis [62, 63]. CC chemokine receptor 4 is expressed on PLTs, and when bound to CCL17, it induces PLT activation and aggregation [64]. To further investigate whether these proteins are clinically relevant in established breast cancers, we performed microdialysis in women before surgery. Seven out of the 11 proteins associated with PLT activation that were upregulated in dense breasts were also upregulated in ER^+^ breast cancer, indeed suggesting that these proteins are associated with a pro-tumorigenic microenvironment.

Beyond their hemostatic functions, PLTs exert substantial influence on cancer progression through the release of inflammatory and pro-angiogenic proteins as well as proteases remodeling the tissue into a pro-tumorigenic microenvironment favoring malignant cell expansion [65]. PLT may be inactivated by NSAIDs, including low-dose ASA, via biochemical inactivation of cyclooxygenases (COX). NSAIDs have been of interest for cancer prevention and therapy, mainly by their anti-inflammatory actions via inhibition of COX. However, large prospective cohort studies failed to find an association between NSAID use and breast cancer risk [66, 67]. Procoagulant activities of PLTs can also be induced by interactions with the extracellular matrix (ECM), and it has been shown that increased tissue stiffness promotes PLT activation [68]. Thus, despite biochemical inhibition, PLTs may still, however, undergo activation via mechanosensitive pathways. Indeed, that is supported by our post hoc exploratory analyses from a randomized trial where postmenopausal women received low-dose ASA versus no treatment for six months [28]. None of the proteins associated with PLT activation with the highest correlation coefficient with breast density were decreased after low-dose ASA. However, we have previously reported that, in dense breast tissue, 20 out of 92 analyzed inflammatory proteins were significantly decreased in addition to blood perfusion alterations after low-dose ASA treatment, indicating biological effects of ASA in this context [28]. Thus, it is unlikely that the observed PLT related protein profile reported in the present study is driven by classical cyclooxygenase-dependent platelet pathways.

In line with these data, it has previously been shown that tissue stiffness per se may induce PLT activation without biochemical pathways with ligand binding to cell surface receptors [68, 69]. To further investigate the role of tissue stiffness on PLT activation, we set up a 3D model with tunable stiffness in ranges corresponding to normal human breasts with different densities. Our in vivo data was consistent with stiffness-dependent effects observed under controlled conditions. A matrix stiffness of 0.85 kPa, resembling dense breasts, promoted a procoagulant PLT phenotype rather than inducing uniform activation. Increased stiffness consistently enhanced Annexin V exposure, indicating phosphatidylserine surface exposure and the acquisition of a procoagulant PLT phenotype. Phosphatidylserine exposure is critical for thrombin generation and defines the procoagulant PLT phenotype [70]. At 0.85 kPa, the stiffness-induced phosphatidylserine exposure occurred in the absence of simultaneous increases in classical PLT activation markers, as neither PAC-1 binding nor α-granule release was increased. This dissociation suggests that matrix stiffness promotes a procoagulant PLT state without inducing full PLT activation, consistent with emerging concepts of PLT functional heterogeneity [71]. In line with this concept, previous flow cytometry studies have shown that activated PLTs can be divided into distinct subpopulations with divergent functional profiles. Normal-sized PLTs mainly display an aggregatory phenotype, with high levels of active αIIbβ3 and intact mitochondria, whereas smaller PLTs and PLT-derived fragments predominantly exhibit exposure of procoagulant phosphatidylserine [72, 73]. Activation of PAR1 signaling increased Annexin V exposure under stiff conditions, whereas inhibition of ROCK, a key regulator of actomyosin contractility [74], reduced overall procoagulant activity, supporting a role for cytoskeletal tension in PLT responses to mechanical cues. Although αIIbβ3 activation, as assessed by PAC-1 binding, was unchanged, integrin-dependent mechanosensitive signaling may still contribute through outside-in signaling and cytoskeletal coupling rather than altered ligand affinity, as previously reported [75]. The increase of DAB2 in human dense breast tissue shown in our present study may indeed suggest a mechanosensitive activation of PLT in vivo, as DAB2 has been shown to be a regulator of outside-in signaling independently of classical integrin activation [76]. Together, these observations suggest that PLT responses to matrix stiffness indeed are driven by mechanotransduction pathways that differ from those driving classical secretory or aggregatory activation. Importantly, while these findings support a role for stiffness-dependent, cytoskeleton-associated PLT responses, they do not exclude contributions from other established mechanosensitive pathways in the breast, including malignant phenotypical transitions of epithelial cells and increased PI3K signaling [77, 78]. We would like to emphasize that the induction of PLT activation was readily detected in a relatively modest stiffness increase of 0.85 kPa, representing normal breast tissue with high mammographic density. This modeling does not represent established breast cancer, where the stiffness is as high as 4–10 kPa [79].

Up to 30% of women aged 40–50 years harbor DCIS, whereas fewer than 1% in the same age group develop invasive breast cancer [10]. Moreover, the prevalence of DCIS in postmenopausal women does not differ according to nondense breast area [5]. These observations suggest that, once atypical epithelial cells arise in the mammary gland, the microenvironment is a key determinant of progression from in situ lesions to invasive, clinically significant breast cancer. Our data suggests that, in addition to the increased levels of pro-inflammatory and angiogenic proteins observed in dense breast tissue, PLT activation via mechanosensitive pathways may represent another mechanism contributing to a pro-tumorigenic microenvironment facilitating cancer progression in dense breasts.

## Conclusions

In summary, our findings demonstrate that dense breast tissue, in addition to immune-related pathways, exhibits a distinct molecular profile suggesting a PLT activation protein profile similar to that found in established ER^+^ breast cancer. In vitro experiments corroborated the in vivo data by showing that tissue stiffness per se indeed contributes to the activation of PLTs. These findings highlight novel opportunities for prevention and treatment of breast cancer by targeting mechanosensitive pathways in PLTs.

### Strengths and limitations of the study

A major strength of this study is the in situ sampling of the extracellular microenvironment in live human breast tissue which, in combination with sampling from abdominal s.c. fat, enables breast-specific analysis of extracellular soluble proteins. The combination with targeted proteomic profiling, covering more than 1,100 proteins, provides a comprehensive characterization of extracellular signaling. The inclusion of well-defined clinical cohorts, nondense and dense breasts, an ASA-treated group, and ER^+^ breast cancer enhances the translational relevance. The quantitative assessment of breast density using MRI further strengthens the relevance of the nondense or dense cohort. Importantly, integration with a 3D in vitro model with tunable stiffness supports a mechanistic link between tissue stiffness and platelet activation.

Limitations include the observational nature of the human data, without any causal inference and the relatively small cohorts. Another limitation is that the analyses are restricted to the extracellular proteome. Additionally, the breast density studies include only healthy postmenopausal women with BMIs within the normal range, which may limit generalizability to premenopausal populations or postmenopausal women with BMIs outside the normal range or with intercurrent diseases. Finally, the 3D model represents a simplified system that cannot fully recapitulate the complexity of an in vivo microenvironment.

## Supplementary Information

Supplementary Material 1.

Supplementary Material 2.

Supplementary Material 3.

## Data Availability

Data is available upon reasonable request.

## Abbreviations

PLT: Platelet
PLTs: Platelets
ER: Estrogen-receptor
ASA: Acetylsalicylic acid
DCIS: Ductal carcinoma in situ
Pa: Pascal
MRI: Magnetic resonance imaging
BI-RADS: Breast Imaging Reporting and Data System
LTF: Lean tissue fraction
PEA: Proximity extension assay
NPX: Normalized protein expression
ACD: Acid–citrate–dextrose
DMEM: Dulbecco’s Modified Eagle Medium
FBS: Fetal bovine serum
PRP: PLT-rich plasma
HA: Hyaluronic acid
BCN: Bicyclo[6.1.0]non-4-yne
SPAAC: Strain-promoted azide–alkyne cycloaddition
KRG: Krebs–Ringer–Glucose
FDR: False discovery rate
GO: Gene ontology
BMI: Body mass index
LOD: level of detection
ECM: Extracellular matrix
NSAID: Non-steroidal anti-inflammatory drug
COX: Cyclooxygenases
