# Supplementary material for: Tissue-stiffness–driven platelet activation emerges as a potential target for breast cancer prevention and treatment
Source: Cell Commun Signal. 2026 Aug 6;24:429. doi: 10.1186/s12964-026-03138-x (PMC13445922; doi:10.1186/s12964-026-03138-x)
Supplement: Supplementary file 1 [file 12964_2026_3138_MOESM1_ESM.docx]

**Table S1.** Characteristic of women with dense or nondense breasts. All values are

median ± 25-75 percentile

|  | Dense  (n = 21) | Nondense  (n = 20) | *P*-value | Statistical method |
| --- | --- | --- | --- | --- |
| Age (years) | 64 (60-70) | 66 (62-70) | 0.4 | Mann-Whitney |
| Body Mass Index (kg/m^2^) | 24.5 (22-27) | 25 (24-28) | 0.2 | Mann-Whitney |
| Breast estradiol (pmol/L) | 38 (28-48) | 36 (28-44) | 0.9 | Mann-Whitney |
| Breast Density (LTF %) | 51 (28-64) | 2.6 (1.4-4.6) | <0.0001 | Mann-Whitney |

**Table S2.** Characteristic of women included in the ASA trial. All values are median ± 25-75 percentile

|  | ASA  (n = 25) | No treatment  (n = 24) | *P*-value | Statistical method |
| --- | --- | --- | --- | --- |
| Age (years) | 65 (55-73) | 66 (56-73) | 0.3 | Mann-Whitney |
| Body Mass Index (kg/m^2^) | 23 (19-30) | 24 (19-27) | 0.4 | Mann-Whitney |
| Breast Density before treatment (LTF %) | 59 (36-92) | 60 (40-90) | 0.8 | Mann-Whitney |

**Table S3**. Characteristics of patients subjected to intratumoral

microdialysis. All cancers were HER-2 negative.

ER=estrogen receptor, PR=progesterone receptor.

| Patient | Age | Tumor size  (mm) | Grade (NHG) | ER (%) | PR (%) |
| --- | --- | --- | --- | --- | --- |
| 1 | 70 | 22 | 2 | >50 | >50 |
| 2 | 68 | 24 | 2 | >50 | >50 |
| 3 | 52 | 25 | 3 | >50 | 10-50 |
| 4 | 78 | 28 | 2 | >50 | >50 |
| 5 | 78 | 19 | 2 | >50 | >50 |
| 6 | 62 | 21 | 2 | >50 | >50 |
| 7 | 61 | 25 | 2 | >50 | >50 |
| 8 | 48 | 30 | 2 | >50 | >50 |
| 9 | 73 | 30 | 2 | >50 | <5 |
| 10 | 57 | 27 | 1 | >50 | >50 |
| 11 | 66 | 60 | 2 | >50 | >50 |
| 12 | 69 | 22 | 2 | >50 | 10-50 |
