# Supplementary material for: Tissue-stiffness–driven platelet activation emerges as a potential target for breast cancer prevention and treatment
Source: Cell Commun Signal. 2026 Aug 6;24:429. doi: 10.1186/s12964-026-03138-x (PMC13445922; doi:10.1186/s12964-026-03138-x)
Supplement: Supplementary file 2 [file 12964_2026_3138_MOESM3_ESM.docx]

Supplementary data

**Tissue-stiffness–driven platelet activation emerges as a potential target for breast cancer prevention and treatment**

^1^Menikae K. Heenkenda, ^1,2^Xingyue Wang*,* ^1, 2^Wen Zhong, ^1,3^Annelie Abrahamsson, ^4^Nina Reustle, ^4^Daniel Aili, ^5,6,7,8^Peter Lundberg, ^1^Tomas L. Lindahl, and ^*1,3^Charlotta Dabrosin

^1^Department of Biomedical and Clinical Sciences, Linköping University, Linköping, Sweden

^2^Science for Life Laboratory, Linköping University, Linköping, Sweden

^3^Clinical Department of Oncology, Region Östergötland, Linköping Sweden

^4^ Laboratory of Molecular Materials, Division of Biophysics and Bioengineering, Department of Physics, Chemistry and Biology, Linköping University, Linköping, Sweden

^5^Clinical Department of Medical Radiation Physics, Region Östergötland, Linköping, Sweden

^6^Center for Medical Image Science and Visualization (CMIV), Linköping University, Linköping, Sweden

^7^Clinical Depart of Radiology in Linköping, Region Östergötland, Linköping, Sweden

^8^Department of Health, Medicine, and Caring Sciences, Linköping University, Linköping, Sweden

**Schematic illustration of the microdialysis procedure in breast tissue in women.**

**Protein network analyses**

Signaling pathways analyses without considerations of breast tissue specific alterations in postmenopausal dense vs. nondense breast tissue Two different database sources for functional analyses, including the Reactome pathway analysis and GO enrichment analysis (Biological Process) were used.

***Figure S1A-B. Reactome Pathway analysis in postmenopausal dense vs. nondense breasts***

Network plot of the top 5 enriched pathways from the Reactome pathway analysis and a dotplot of all enriched pathways from the Reactome pathway analysis.

***Figure S1C-D GO enrichment analysis in postmenopausal dense vs. nondense breasts***

Network plot of the top 5 enriched terms from the GO analysis and a dotplot of the top 30 GO terms.

**Identification of appropriate matrix ligand**

To evaluate the influence of matrix ligand composition on platelet (PLT) behavior under dense-matrix conditions, estrogen receptor positive (ER+) breast cancer cells MCF-7 and PLTs were co-cultured in 3D hydrogels. Cyclic RGD (Arg–Gly–Asp) (cRGD-Az) was synthesized as previously described and covalently incorporated into the hydrogel network at a final concentration of 100 µM (1). In parallel, collagen type I (0.1 mg/mL) or fibrinogen (50 µg/mL) was added to the hydrogel precursor solution without covalent functionalization.

In cRGD- and collagen type I-containing hydrogels, CD41⁺ PLTs show phalloidin overlap consistent with cytoskeletal reorganization and form small aggregates. In contrast, fibrinogen-containing hydrogels support stable PLT association with a distinct CD41 signal and minimal aggregation. Scale bar, 10 µm.

cRGD-functionalized hydrogels exhibited a reduced number of PLTs associated with the matrix, whereas collagen type I promoted pronounced PLT activation. In contrast, fibrinogen-containing hydrogels supported stable PLT association as shown in representative images in Supplementary Figure S2.

***Figure S2. Matrix ligand selection for PLT activation.***

Representative confocal images of MCF-7 cells co-cultured PLTs in 3D HA-BCN hydrogels after 2h. PLTs are identified by CD41-AF488 (green) and MCF-7 cells by F-actin staining using rhodamine-phalloidin (red). Hydrogels contained different matrix ligands: (a) cRGD covalently functionalized to the hydrogel network (100 µM); (b) collagen type I (0.1 mg/mL); (c) fibrinogen (50 µg/mL). Scale bar = 10 µm.

**Identification and quantification of classical PLT markers**

Human PLTs were co-cultured with estrogen receptor–positive MCF-7 breast cancer cells in 3D HA-BCN hydrogels of low (0.2 Pka) or high (0.85 kPa) matrix stiffness and analyzed after 2 h or 24 h. Classical PLT activation was assessed by surface expression of PAC-1 and P-selectin, Supplementary Figure S3.

***Figure S3. Matrix stiffness did not alter classical PLT activation markers in co-culture with breast cancer cells.***

**(A)** PAC-1-positive PLTs after 2 h of co-culture.
**(B)** P-selectin-positive PLTs after 2 h of co-culture.
**(C)** PAC-1-positive PLTs after 24 h of co-culture.
**(D)** P-selectin-positive PLTs after 24 h of co-culture.

No significant differences were detected between stiffness conditions. Data are shown as mean ± SD; *n* = 4; Student’s t-test; ns, not significant.

**Identification of tissue residing PLTs**

Formalin-fixed, paraffin-embedded normal breast tissue was cut into 4-μm sections. Deparaffined sections were boiled at +98°C for 15 min in Diva Decloaker (Biocare Medical, Pacheco, CA, Cat# DV2004MX) for antigen retrieval, blocked 20 min with Rodent Block M (Biocare Medical, Cat# RBM961G) and stained with mouse anti-human CD41a (BD Biosciences Cat# 333147, RRID:AB_2868648, 1:5) and Hoechst (Sigma, Cat# 33342, 1:5000). Antibodies were diluted in Antibody Diluent with Background Reducing reagent (Agilent/DAKO, Glostrup, Denmark, Cat# S302281-2). Olympus BX43 light/fluorescence microscope, excitation filter BP360-370 and BP460-495, equipped with an Olympus DP74 3CCD mode camera was used for visualization and for images CellSens imaging software version 1.15 (Olympus CellSens Software, RRID:SCR_014551) was used.

***Figure S4. PLTs reside within normal breast tissue***

Cell nuclei in blue (Hoechst). White arrows indicate CD41a positive PLTs scattered in the tissue.

Scale bar = 50 µm

**Reference:**

1. Abrahamsson A, Boroojeni FR, Naeimipour S, Reustle N, Selegard R, Aili D, et al. Increased matrix stiffness enhances pro-tumorigenic traits in a physiologically relevant breast tissue- monocyte 3D model. Acta Biomater. 2024;178:160-9.
